# The relationship between vaccine hesitancy and health literacy in pregnant women: a cross-sectional study *

**DOI:** 10.1186/s12905-024-03148-2

**Published:** 2024-06-21

**Authors:** Kübra Çetin, Seda Cangöl Sögüt

**Affiliations:** 1 Midwife, Tekirdag Dr. İsmail Fehmi Cumalıoğlu City Hospital, Tekirdağ, Turkey; 2 Department of Midwifery, Faculty of Health Sciences, Çanakkale Onsekiz Mart University, Çanakkale, Turkey

**Keywords:** Pregnant, Health literacy, Anti-vaccination

## Abstract

**Background:**

Pregnancy; It is an important process that directly affects the mother and the fetus, where women benefit more from health services and the need for health-related decision-making and information increases. It is very important to determine and improve the health literacy level of these women. The study determined the relationship between vaccine hesitancy and health literacy in pregnant women.

**Methods:**

It is a cross-sectional type of research. The research was carried out in a state hospital. The online form was sent to 230 pregnant women. Ethics committee, institution and scale permissions were obtained for the study. The data of the study were collected online by using the questionnaire prepared by scanning the literature, the Anti-Vaccination Scale and the Health Literacy Scale. Statistical analyzes of the data were performed using the SPSS version 25 program.

**Results:**

The Vaccine Hesitancy Scale score of the pregnant women was 55.53 ± 10.15, whereas their Health Literacy Scale score was 98.57 ± 21.48. Health literacy was associated with the sociodemographic and obstetric characteristics of the pregnant women. Educational status, economic status, place of residence, and family structure were associated with vaccine hesitancy.

**Conclusions:**

It was determined that there was a negative correlation between the anti-vaccination scale scores of the pregnant women and the health literacy scale scores. As a result, it was determined that the anti-vaccination level of the pregnant women was moderate and the health literacy level was sufficient. It can be recommended to provide appropriate trainings and counseling to target groups, which will increase the health literacy level of pregnant women.

**Supplementary Information:**

The online version contains supplementary material available at 10.1186/s12905-024-03148-2.

## Introduction

Health literacy is the capacity that determines people’s ability to access, comprehend and use accurate information that supports their appropriate health status and is sustainable throughout their lives [1]. Pregnancy is an important process directly affecting the mother and fetus, and during this process, women benefit more from health services, and their need for health-related decision making and information increases. This is an important opportunity to determine and improve the health literacy level of women [2].

Considering various studies in the world, in a cross-sectional study conducted in Germany (2017), it was determined that 54.3% of individuals had limited health literacy [3]. It was reported that 29% of people in the Netherlands had low health literacy, with the lowest health literacy rates being Bulgaria (37%) and Spain (42%) [4]. In a study conducted in China, it was determined that 81% of individuals had a low level of health literacy [5].

It has been found that 69.4% of individuals in Turkey have insufficient and problematic health literacy levels [6]. In the other study, it was determined that 55.4% of them had insufficient health literacy level, 22.2% of them had adequate and excellent level of health literacy [7].

Vaccination is the simplest, safest and most effective way to prevent infectious diseases. They build up resistance against certain infections by using the body’s natural defenses. Vaccines save about 2–3 million lives each year [8]. Although vaccine hesitancy has accelerated in the world since 1990, the concept of vaccine hesitancy came to the fore with the vaccination studies of Edward Jenner in England in 1796. Vaccination began to be widespread in the 1800s [9]. According to WHO, “vaccine hesitation” means delaying in accepting the administration of some vaccines or not allowing the administration of some vaccines even though vaccine availability is possible. “vaccine opposition” is the situation in which individuals do not voluntarily receive all vaccines [10].

Although great advances were made in vaccination in the past century, vaccine-preventable diseases are re-emerging due to vaccine hesitancy and remain a major threat to society [11]. Poor health literacy has been associated with less adoption of protective behaviors such as immunizations [12]. It is thought that individuals with a high level of health literacy will be able to get information about vaccination from the right sources and have an important place in the fight against vaccine opposition, which has become a major threat to the society [13].

Women’s health behaviors are decisive in raising and improving the health standards of the society. Pregnancy is a critical period when women use basic health services more frequently and become more open to learning [14]. Maternal health literacy level affects the ability of pregnant women to make the best decisions for themselves and their babies [15]. It was determined that health literacy training given to pregnant women increased their compliance with pregnancy, general self-efficacy and health literacy level [16].

It is important to determine the anti-vaccination opposition in pregnant women and the concept of health literacy, which increases its importance day by day. Community health nurses, midwives and social workers are key in this regard. Studies examining the relationship between anti-vaccination and health literacy in pregnant women are limited. Its aim is to examine the relationship between anti-vaccination and health literacy in pregnant women.

## Methods

### Participants and procedures

The population of this cross-sectional study consisted of pregnant women who applied to a city hospital in western Turkey. In the study, the calculation (d-value) method developed by Cohen was used to calculate the effect size in order to examine the relationship between vaccine hesitancy and health literacy in pregnant women. In the light of academic studies, whose d value, which is the effect size index, was reported, the “G.Power-3.1.9.2” program was used in the sample calculation (d = 0.25, 1-α=%95, 1-β=%95), and the number of participants was calculated as 197 pregnant women [13, 17]. The sample consisted of 230 pregnant women who agreed to participate in the study. The data were collected by applying an online form to 230 pregnant women who agreed to participate in the study. The online form was collected by delivering it to the pregnant women through the pregnancy school group. The pregnant women who did not have a Turkish comprehension problem were included in the sampling inclusion criteria. The study will take place between April 2021 and February 2022. The vaccine names included in the study are flu vaccine, tetanus-diphtheria-pertussis vaccine during pregnancy, hepatitis B vaccine during pregnancy and rabies vaccine. The study was conducted in a hospital due to easier access to pregnant women.

### Measures

The data were collected by the researchers using a questionnaire, the “Vaccine Hesitancy Scale”; and the “Health Literacy Scale” were used.

#### Data collection form

The survey, which consists of 2 parts, includes 25 questions in total. In the first part, there are 18 questions including “socio-demographic characteristics” (age, gender, educational status, family type, number of pregnancies, etc.), while in the second part, there are 7 questions in which the data about “vaccine and health literacy” (vaccination status, where they got information such as getting vaccinated with a doctor’s recommendation, etc.) were collected. The questions were formed in line with the literature [13, 18, 19]. In the questionnaire, participants’ gestational week, 1st, 2nd week. and 3rd trimester.

#### Vaccine hesitancy scale

The Vaccine Hesitancy Scale (VHS) was developed by Kılınçarslan et al. [20] to measure the level of vaccine hesitation. This scale is a 5-point Likert scale. The total score can vary between 21 and 105. A higher score on the scale means that participants have higher hesitations about vaccination [20]. (Appendix-1). The Cronbach Alpha (a) internal consistency value of this study was determined as 0.87.

#### Health literacy scale

It was developed as 47 items by Sørensen et al. [21] to measure the health literacy levels of individuals, and simplified as 25 items by Toçi et al. [22]. The Turkish validity and reliability analyzes of the Health Literacy Scale (HLS) were performed by Aras and Temel-Bayık [23]. The scale, which consists of 25 items and four subscales, is answered in a 5-point likert structure. All items of the scale have a positive structure, and there is no reverse item. The higher the score, the higher the level of health literacy [23]. (Appendix-2). The Cronbach Alpha (a) coefficient of this study was found to be 0.97.

### Statistical analysis

Analyzes were calculated with SPSS version 25 program. Kolmogorov-Smirnov test was used to test the normality of scores obtained from a continuous variable. In addition to descriptive statistical methods while evaluating the study data, Independent Sample t-Test and One-Way ANOVA (Variance) Analysis were used to test the quantitative difference between groups. Qualitative variables in the research are frequency (n, %); continuous variables were presented as mean and standard deviation. Multiple comparisons were calculated with Scheffe test in groups where the difference in the ANOVA test results was significant. The level of relationship between two continuous variables was evaluated with the Pearson correlation test. The results were calculated at the 95% confidence interval, and the significance was calculated at the *p* < 0.05 level.

## Results

230 pregnant women were included in the study. The mean age of the pregnant women was 28.20 ± 6.08; 63.5% were under the age of 30; and 97% were married. According to their education level, 24.8% of the pregnant women were primary school graduates, 33.9% were secondary school graduates, and 41.3% were higher education graduates. According to their economic status, it was determined that 35.2% of the pregnant women’s income did not cover their expenses, that 47.4% of the pregnant women’s income was equal to their expenses, and that 17.4% of the pregnant women’s income was more than their expenses. 23.5% of the pregnant women were actively working in a job,; 93% were living in a district or province; 83.5% had a social security; 84.8% had a nuclear family structure; and 7% had a diagnosed chronic disease. It was determined from the available data that 7% of them used a drug regularly, that 25.2% used cigarettes, and that 7.4% used alcohol. (Table 1).

**Table 1:** Sociodemographic characteristics of pregnant women (*n* = 230)

Variables	Category	Number(*n*)	%	Mean ± SD	Minimum-Maximum
Age	All	230	100,0	28,20 ± 6,08	17–46
Age group	< 30	146	63,5		
	≥ 30	84	36,5		
Marital status	married	223	97,0		
	single	7	3,0		
Education	Elementary Education	57	24,8		
	Secondary education	78	33,9		
	High education	95	41,3		
Working status	Yes	54	23,5		
	No	176	76,5		
Place of residence	Village	16	7,0		
	District	58	25,2		
	city	156	67,8		
Income status	Income less than expenses	81	35,2		
	Income equal to expenses	109	47,4		
	Income higher than expenses	40	17,4		
Social security	Yes	192	83,5		
	No	38	16,5		
Family type	Extended family	35	15,2		
	Nuclear family	195	84,8		
Chronic disease	Yes	16	7,0		
	No	214	93,0		
Smoking	Yes	58	25,2		
	No	172	74,8		
Alcohol use	Yes	17	7,4		
	No	213	92,6		
Regular use of medication	Yes	16	7,0		
	No	214	93,0		

When the total and sub-dimension scores of the pregnant women on the HLS were examined, it was determined that the total score was 98.57 ± 21.48. When the total score of the pregnant women on the VHS was examined, it was determined that the total score was 55.53 ± 10.15.

It was determined that the HLS scores differed statistically significantly according to marital status, education level, place of residence, and economic status. It was determined that the level of health literacy increased in the married participants, those with a high education level, those living in cities, and those with a high economic status (*p* < 0.05). It was determined that the health literacy levels of the pregnant women, who had 3 or more pregnancies and had 3 or more births, were higher. (*p* = 0.003; *p* = 0.001).

It was determined that the VHS scores of the pregnant women differed statistically significantly according to education level, place of residence, economic level, and family structure, and that the anti-vaccination rate of the pregnant women living in the city was lower (*p* < 0.05).

It was determined that 77% of the pregnant women had their vaccinations completely according to the pregnancy calendar, that 20.9% had the flu vaccine in the last 1 year, and that 56.1% received information about vaccines from health staff. 58.7% stated that the information they obtained about vaccines was positive, while 44.8% stated that the information obtained from the written and visual media about vaccines never changed their opinion about the vaccine. 73% of the pregnant women stated that they could get the tetanus vaccine upon the recommendation of a doctor, whereas 65.2% stated that they could get the hepatitis-B vaccine. It was determined from the available data that 10% of the pregnant women thought that it was inconvenient to have the rabies vaccination in case of suspected animal bites during pregnancy. (Table 2).

**Table 2:** Vaccination- and health literacy-related characteristics of pregnant women (*n* = 230)

Variables	Category	Number(*n*)	%
Status of having all vaccinations according to the vaccination calendar	Yes-All	177	77,0
	No-Some vaccines	53	23,0
Seasonal flu vaccination status within 1 year	Yes	48	20,9
	No	182	79,1
Source of information about vaccines	Healthcare personnel	129	56,1
	Internet/social media	46	20,0
	Written or visual media	45	19,6
	scientific articles	10	4,3
Positive information about vaccines	Yes	135	58,7
	Partially	86	37,4
	No	9	3,9
Change of opinion about vaccines as a result of information obtained from television, radio or the internet	Yes, rarely	105	45,7
	Yes, often	22	9,6
	No, never	103	44,8
Consideration of getting the tetanus-diphtheria-pertussis vaccine during pregnancy with doctor’s recommendation	Yes	168	73,0
	No	15	6,5
	I don’t know	47	20,4
Consideration of getting the hepatitis B vaccine during pregnancy with doctor’s recommendation	Evet	150	65,2
	No	22	9,6
	I don’t know	58	25,2
The state of thinking that it is inconvenient to have the rabies vaccine in case of suspected animal bites during pregnancy.	Yes	23	10,0
	No	69	30,0
	I don’t know	138	60,0

It was determined that the mean VHS scores of the pregnant women who were regularly vaccinated according to the immunization schedule were statistically significantly lower (t = 5.334; *p* < 0.001).

It was determined that the VHS scores of the pregnant women differed statistically significantly according to the source of information about vaccines (F = 9,661; *p* < 0.001). In the subgroup analyzes, it was found out that this difference was caused by the pregnant women whose vaccine information source was scientific publications on vaccines. From this finding, it was determined that the anti-vaccination resistance of the pregnant women who had information about the vaccine from scientific publications was lower.

It was found that the mean VHS score of the pregnant women, who thought that the information they obtained about vaccines was positive, was statistically significantly lower (t = 4.919; *p* < 0.001). It was found that the mean HLS scores of the pregnant women, who changed their minds about vaccines as a result of the information they obtained from television, radio or the internet, were found to be statistically significantly higher (t = 4.834; *p* < 0.001). It was found that the mean VHS score of the pregnant women, who stated that they could get the tetanus-diphtheria-pertussis vaccine during pregnancy upon the recommendation of a doctor, was statistically significantly lower (t = 4.847; *p* < 0.001). It was determined that the mean HLS scores of the pregnant women, who stated that they could get the hepatitis-B vaccine during pregnancy upon the recommendation of a doctor, were statistically significantly lower (t = 3.570; *p* < 0.001). (Table 3)

**Table 3:** Vaccine hesitancy scale (VHS) mean scores of pregnant women according to vaccination- and health literacy-related characteristics (*N* = 230)

	Vaccine Hesitancy Scale			
Variables	Mean ± SD	t/F	*p*	Fark**
**Status of having all vaccinations according to the vaccination calendar**		*5,334* ^*a*^	< 0,001*	
Yes-All	53,69 ± 9,98			
No-Some vaccines	61,70 ± 8,13			
**Seasonal flu vaccination status within 1 year**		*1,648* ^*a*^	*0,101*	
Yes	53,40 ± 8,00			
No	56,10 ± 10,59			
**Source of information about vaccines**		*9,661* ^*b*^	< 0,001*	_***f=4<2,3***_
Healthcare personnel^1^	53,97 ± 10,44			
Internet/social media^2^	56,52 ± 8,13			
Written or visual media^3^	61,13 ± 7,33			
scientific articles^4^	46,00 ± 13,42			
**Positive information about vaccines**		*4,919* ^*a*^	< 0,001*	
Yes	52,90 ± 9,96			
Partially/No	59,27 ± 9,24			
**Change of opinion about vaccines as a result of information obtained from television, radio or the internet**		*4,834* ^*a*^	< 0,001*	
Yes, rarely/often	58,32 ± 8,80			
No, never	52,11 ± 10,68			
**Consideration of getting the tetanus-diphtheria-pertussis vaccine during pregnancy with doctor’s recommendation**		*4,847* ^*a*^	< 0,001*	
Yes	53,65 ± 9,43			
No, I don’t know	60,63 ± 10,36			
**Consideration of getting the hepatitis B vaccine during pregnancy with doctor’s recommendation**		*3,570* ^*a*^	< 0,001*	
Yes	53,83 ± 9,11			
No, I don’t know	58,73 ± 11,24			
**The state of thinking that it is inconvenient to have the rabies vaccine in case of suspected animal bites during pregnancy**		*1,580* ^*a*^	*0,116*	
Yes	58,70 ± 11,55			
No, I don’t know	55,18 ± 9,95			

a(t): Independent Sample t-test; b(F): ANOVA(Variance) Analysis, **: Schefft test

A statistically significant and negative correlation was found between the VHS scores of the pregnant women and their HLS total and sub-dimension scores (*p* < 0.001). From this finding, it was determined that as the health literacy level of the pregnant women decreased, the anti-vaccination attitude increased.

A statistically significant and positive correlation was found between the HLS total score and the HLS sub-dimension scores (*p* < 0.001). From this finding, it was determined that as the HLS sub-dimension scores increased, the total scale score also increased (Table 4).

**Table 4:** Level of relationship between scale (VHS and HLS) scores

		VHS	Access to Information	Understanding Information	Appraisal/Assessment	Application/Use
HLS **- Access to Information**	*r*	-0,398				
	*p*	< 0,001*				
HLS **- Understanding Information**	*r*	-0,420	0,826			
	*p*	< 0,001*	< 0,001*			
HLS **- Appraisal/Assessment**	*r*	-0,389	0,764	0,864		
	*p*	< 0,001*	< 0,001*	< 0,001*		
HLS **- application/use**	*r*	-0,392	0,697	0,750	0,842	
	*p*	< 0,001*	< 0,001*	< 0,001*	< 0,001*	
HLS **-Total**	*r*	-0,433	0,888	0,943	0,953	0,886
	*p*	< 0,001*	< 0,001*	< 0,001*	< 0,001*	< 0,001*

*:*p* < 0,05; ***r***: Pearson correlation test

## Discussion

It was determined that the general vaccine hesitancy score averages of the individuals participating in our study were 55.53 ± 10.15. Pregnant women are a high-risk group for the effects of Covid-19 infection [24, 25]. In a study investigating the factors affecting Covid-19 vaccination in pregnant women, it was reported that only 51.5% of pregnant women accepted to be vaccinated, and that the determined rate was lower than the general population vaccination rate [25].

In the study, it was determined that one of the factors affecting vaccine hesitancy was the level of education. It was determined that the anti-vaccination rate was higher in the primary school graduates. In the study conducted by Çınar et al. [26] the tetanus immunization status of pregnant women, their frequency, their approach to the tetanus vaccine and the factors affecting it were determined, and it was reported that high school or higher education increased vaccination. Polat et al. [25] showed that as the level of education increased, the rate of vaccination also increased. In the study conducted by Afolabi et al. [27], it was concluded that pregnant women with a lower education level were more likely to hesitate in taking the hepatitis B vaccine. It is thought that as the education level of pregnant women increases, access to the right information from the right source increases. The study supports the literature.

In the study, it was determined that the vaccine opposition of the pregnant women who thought that their knowledge about vaccines was positive was lower. In the study of Dağdeviren et al. [28] when the reasons for vaccine opposition in pregnant women were questioned, 46% reported that they did not know that they should be vaccinated, 12.5% thought that the vaccine was not protective, and 2.3% reported that they had concerns about the content of the vaccine. In a study, it was reported that fear was the leading reason for pregnant women not to have the tetanus vaccine [26]. In the study conducted on pregnant women, it was reported that the information conveyed about the safety and importance of vaccination increased the intake of tetanus, diphtheria and pertussis (Tdap) vaccines in pregnant women [29]. In a study conducted in China, it was determined that individuals who received negative information about the Covid-19 vaccine and had doubts about the source of information were more likely to experience vaccine hesitancy [30]. According to a study conducted in Kelantan, participants had concerns such as doubts about the safety of the Covid- 19 vaccine (51.6%), doubts about its effectiveness (50.7%) and fear of adverse effects (61.1%) [31]. The fact that pregnant women have sufficient and positive knowledge about vaccines affects the level of anti-vaccination.

In a study, it was concluded that the second most common reason for individuals to experience vaccine hesitancy was the information they heard from the media and the internet. 25% of individuals participating in that study stated that they heard that the vaccine was harmful from the media and the internet [32]. In a study, it was seen that individuals who used medical websites as a source of information had a more positive attitude towards vaccines than individuals who used social media [33]. In this study, it was found that the pregnant women who changed their minds about vaccines as a result of the information they received from television, radio or the internet had higher anti-vaccination. The available literature supports the findings of this study. Non-scientific data disseminated through the media and social media play an important role in vaccine rejection and opposition.

In another study, it was determined that individuals who received information about vaccines from healthcare professionals were more likely to consider having their children vaccinated [34]. It was concluded that the main reason for women to receive seasonal influenza and pertussis vaccines during pregnancy in Ireland was the recommendation of a doctor [35]. In another study conducted in Italy, the main barriers to vaccination for influenza and pertussis vaccines were determined as not receiving vaccination advice from any healthcare provider (81%) and safety concerns (18%) [36]. Considering the results of the studies, it is thought that vaccination can be increased with the advice of a health professional or doctor.

In society, especially in pandemics, governments should cooperate on increasing vaccination programs [37]. Health education and consultancy services are important to increase immunization knowledge [38]. A study conducted in adults reported that HLS had a positive effect on immunization [39]. In another study, it was found that pregnant women with high HLS levels had lower teratogenic risk perceptions regarding the flu vaccine [2]. The findings of our study support the literature, but it seems that more study results are needed.

In the study in which the immunization knowledge proficiency levels, attitudes and behaviors, and health literacy levels of adults were evaluated, it was determined that there was a positive relationship between the level of health literacy and the level of immunization knowledge and attitudes towards immunization services [13]. In a study conducted in Spain, it was concluded that women with high health literacy levels were more likely to refuse immunization [40]. In another study, it was concluded that as the health literacy level increased, the vaccine hesitancy decreased [41]. Another study states that increasing education against vaccine refusal and incorporating motivational interviewing skills are the first steps towards increasing mothers’ vaccine acceptance [42]. In another study, there was a relationship between internet decision-making and COVID-19 vaccine hesitancy in pregnant women. Health professionals, information specialists and librarians emphasize that they should direct people to reliable sources about vaccines [43]. In this study, a statistically significant and negative correlation was found between the VHS scores of the pregnant women and their HLS total and sub-dimension scores. The results of this study support the literature, but it is known as the first study to examine vaccine hesitancy and health literacy in pregnant women. It seems that more academic studies are needed.

No difference was found between pregnant women’s opposition to vaccination according to pregnancy characteristics (gestational week, number of pregnancies, number of live births and miscarriage). In a study, it was reported that the vaccination rates of nulliparous pregnant women were higher [44]. In another study, it was determined that multiparous people had higher rates of not accepting the vaccine [25]. In a study investigating flu vaccine uptake among pregnant women in Singapore, no difference in vaccine uptake was found according to trimester [45].

## Conclusion

The Vaccine Hesitancy Scale score of the pregnant women was 55.53 ± 10.15, while their Health Literacy Scale score was 98.57 ± 21.48. It was determined that the health literacy level of the pregnant women was sufficient and that the level of vaccine hesitancy was moderate.

It was determined that the HLS total and sub-dimension scores differed statistically significantly according to marital status, education level, place of residence, economic status, and number of pregnancies (*p* < 0.05). It was determined that the level of health literacy increased among those with a high level of education, those living in cities, and those with a high economic status. It was determined that the health literacy levels of the pregnant women who had 3 or more pregnancies and had 3 or more births were lower.

It was determined that the VHS scores of the pregnant women showed statistically significant differences according to education level, place of residence, economic level, and family structure. It was found that the mean VHS scores of the pregnant women who graduated from primary school, those with low economic status, and those with extended family structure were statistically significantly higher. It was determined that the anti-vaccination rate of the pregnant women living in cities was lower, and that there was a negative correlation between the Vaccine Hesitancy Scale scores of the pregnant women and their health literacy scale scores.

In light of the findings, this study is the first known research in the literature examining health literacy and vaccine hesitancy in pregnant women. It was determined that there was a negative relationship between pregnant women’s anti-vaccine scale scores and health literacy scale scores. As a result, it was determined that the pregnant women’s opposition to vaccination was at a moderate level and their health literacy level was sufficient. Training programs about the importance of immunization given to pregnant women by community health nurses will raise awareness in pregnant women. Community health nurses should provide appropriate training and consultancy to the target group, which will increase the health literacy level of pregnant women. Successful educational interventions on vaccine hesitancy and health literacy contribute to pregnant women on how and from which sources they can access accurate information. Community health nurses, midwives and social workers should fight against vaccination in cooperation. Interventional studies on vaccine hesitancy and health literacy in pregnant women are recommended.

### Limitations

This study is the first known research in the literature examining health literacy and vaccine hesitancy in pregnant women. However, the study has some limitations. The first of these is the collection of the data based on the self-reports of the pregnant women. In addition, since the results of the research are related to the sample in which the research was conducted, they cannot be generalized.

### Electronic supplementary material

Below is the link to the electronic supplementary material.

Supplementary Material 1

## Data Availability

The data that support the findings of this study are available from Seda Cangöl Sögüt but restrictions apply to the availability of these data, which were used under license for the current study, and so are not publicly available. Data are how ever available from the authors upon reasonable request and with permission of Seda Cangöl Sögüt.
