# Supplementary material for: The relationship between vaccine hesitancy and health literacy in pregnant women: a cross-sectional study *
Source: BMC Womens Health. 2024 Jun 21;24:361. doi: 10.1186/s12905-024-03148-2 (PMC11191248; doi:10.1186/s12905-024-03148-2)
Supplement: Supplementary file 1 — Supplementary Material 1 [file 12905_2024_3148_MOESM1_ESM.docx]

Appendix-1

**Vaccine Hesitancy Scale**

|  | | Strongly disagree | Disagree | Partially agree | Agree | Strongly agree |
| --- | --- | --- | --- | --- | --- | --- |
| A1 | If everyone gets vaccinated, diseases will decrease. |  |  |  |  |  |
| A2 | Vaccination is an effective method to protect health. |  |  |  |  |  |
| A3 | I trust the vaccines recommended by the government. |  |  |  |  |  |
| A4 | he strongest measure against pandemics is vaccination. |  |  |  |  |  |
| A5 | Vaccination is an important safeguard for our health.. |  |  |  |  |  |
| B1 | I am worried about the side effects of vaccines. |  |  |  |  |  |
| B2 | I'm afraid that vaccines may cause autism or learning disorders |  |  |  |  |  |
| B3 | Vaccines can cause many diseases. |  |  |  |  |  |
| B4 | Vaccination benefits vaccine producers more than people's health. |  |  |  |  |  |
| B5 | Vaccines have as much harm as they have benefits. |  |  |  |  |  |
| B6 | Vaccines contain toxic substances. |  |  |  |  |  |
| C1 | Traditional methods passed down from ancestors provide better protection than vaccines. |  |  |  |  |  |
| C2 | I prefer to get the disease rather than getting vaccinated to gain immunity. |  |  |  |  |  |
| C3 | If I could, I would abolish mandatory vaccination. |  |  |  |  |  |
| C4 | Vaccination should be optional, not mandatory. |  |  |  |  |  |
| C5 | If I could go back to my childhood, I wouldn't get vaccinated. |  |  |  |  |  |
| D1 | I won't get vaccinated because I'm afraid of needles. |  |  |  |  |  |
| D2 | I won't get vaccinated because of my religious beliefs. |  |  |  |  |  |
| D3 | I will not vaccinate my child because vaccines can cause permanent illness. |  |  |  |  |  |
| D4 | My child doesn't need to be vaccinated because other children are vaccinated. |  |  |  |  |  |
| D5 | Vaccination is unnecessary because contagious diseases are rare. |  |  |  |  |  |

Appendix-2

**Health Literacy Scale**

| **ACCESS TO INFORMATION** | I have no difficulty at all | I have some difficulty | I have little difficulty | I have a lot of difficulty | I am unable to do it at all, I have no ability/it's impossible |
| --- | --- | --- | --- | --- | --- |
| 1. Can you find information about diseases? |  |  |  |  |  |
| 2. Are you informed about treatments? |  |  |  |  |  |
| 3. Can you find information about health risks such as smoking, obesity, etc.? |  |  |  |  |  |
| 4. Can you find information on how to stay healthy? |  |  |  |  |  |
| 5. Can you obtain information about healthy foods and how to stay in shape? |  |  |  |  |  |
| **UNDERSTANDING INFORMATION** |  |  |  |  |  |
| 6. Can you understand the explanatory information found on medicine packaging? |  |  |  |  |  |
| 7. Can you understand medical prescriptions? |  |  |  |  |  |
| 8. Can you read brochures providing information about behaviors harmful to health found in pharmacies, hospitals, or doctor's offices? |  |  |  |  |  |
| 9. Can you understand information about dangerous behaviors such as smoking, drug use, drunk driving, etc.? |  |  |  |  |  |
| 10. Can you understand the contents of food labels? |  |  |  |  |  |
| 11. Do you understand the importance of healthy lifestyles? |  |  |  |  |  |
| 12. Can you understand the importance of a healthy environment at home, school, workplace, or in the neighborhood? |  |  |  |  |  |
| **VALUATION / EVALUATION** |  |  |  |  |  |
| 13. Can you discuss medical information with your doctor or pharmacist? |  |  |  |  |  |
| 14. Can you consider the side effects or benefits of treatment options? |  |  |  |  |  |
| 15. Can you decide which medical recommendations are best for you? |  |  |  |  |  |
| 16. Can you identify behaviors harmful to your health? |  |  |  |  |  |
| 17. Can you learn from the unhealthy behaviors of others? |  |  |  |  |  |
| 18. Can you carefully evaluate information about unhealthy behaviors obtained from health professionals, friends, family, or sources such as radio, newspapers, television? |  |  |  |  |  |
| 19. Can you evaluate your health-related habits? |  |  |  |  |  |
| 20. Can you consider the effects and benefits of healthy choices such as healthy eating or exercise? |  |  |  |  |  |
| **APPLICATION / USAGE** |  |  |  |  |  |
| 21. Can you follow the recommendations given by doctors, nurses, or pharmacists? |  |  |  |  |  |
| 22. Can you adhere to the recommendations given by healthcare professionals, such as getting vaccinated, participating in screening programs, or driving safely? |  |  |  |  |  |
| 23. Can you change your unhealthy habits if you want to? |  |  |  |  |  |
| 24. Can you access healthy products (such as natural foods and harmless chemicals)? |  |  |  |  |  |
| 25. Can you use health-related information to your advantage? |  |  |  |  |  |
